# Reevaluating surgery and re-irradiation for locally recurrent pediatric ependymoma—a multi-institutional study

**DOI:** 10.1093/noajnl/vdab158

**Published:** 2021-11-08

**Authors:** David Y Mak, Normand Laperriere, Vijay Ramaswamy, Eric Bouffet, Jeffrey C Murray, Rene Y McNall-Knapp, Kevin Bielamowicz, Arnold C Paulino, Wafik Zaky, Susan L McGovern, M Fatih Okcu, Uri Tabori, Doaa Atwi, Peter B Dirks, Michael D Taylor, Derek S Tsang, Abhishek Bavle

**Affiliations:** 1 Radiation Medicine Program, Princess Margaret Cancer Centre, University Health Network, Toronto, Ontario, Canada; 2 Division of Haematology/Oncology, Hospital for Sick Children, Toronto, Ontario, Canada; 3 Division of Neurosurgery, Hospital for Sick Children, Toronto, Ontario, Canada; 4 Pediatric Hematology/Oncology, Cook Children’s Medical Center, Fort Worth, Texas, USA; 5 Section of Pediatric Hematology/Oncology, University of Oklahoma Health Sciences Center, Oklahoma City, Oklahoma, USA; 6 Section of Pediatric Hematology/Oncology, University of Arkansas for Medical Sciences, Little Rock, Arkansas, USA; 7 Department of Radiation Oncology, MD Anderson Cancer Center, Houston, Texas, USA; 8 Division of Pediatrics, MD Anderson Cancer Center, Houston, Texas, USA; 9 Section of Pediatric Hematology/Oncology, Texas Children’s Cancer Center, Baylor College of Medicine, Houston, Texas, USA; 10 Department of Pathology, University of Oklahoma Health Sciences Center, Oklahoma City, Oklahoma, USA; 11 Children’s Blood and Cancer Center, Dell Children’s Medical Center of Central Texas, Austin, Texas, USA

**Keywords:** ependymoma, pediatric, recurrent, reirradiation, re-resection

## Abstract

**Background:**

The goal of this study was to evaluate extent of surgical resection, and timing and volume of re-irradiation, on survival for children with locally recurrent ependymoma.

**Methods:**

Children with locally recurrent ependymoma treated with a second course of fractionated radiotherapy (RT2) from 6 North American cancer centers were reviewed. The index time was from the start of RT2 unless otherwise stated.

**Results:**

Thirty-five patients were included in the study. The median doses for first radiation (RT1) and RT2 were 55.8 and 54 Gy, respectively. Median follow-up time was 5.6 years. Median overall survival (OS) for all patients from RT2 was 65 months. Gross total resection (GTR) was performed in 46% and 66% of patients prior to RT1 and RT2, respectively. GTR prior to RT2 was independently associated with improved progression-free survival (PFS) for all patients (HR 0.41, *P* = 0.04), with an OS benefit (HR 0.26, *P* = 0.03) for infratentorial tumors. Median PFS was superior with craniospinal irradiation (CSI) RT2 (not reached) compared to focal RT2 (56.9 months; log-rank *P* = 0.03). All distant failures (except one) occurred after focal RT2. Local failures after focal RT2 were predominantly in patients with less than GTR pre-RT2.

**Conclusions:**

Patients with locally recurrent pediatric ependymoma should be considered for re-treatment with repeat maximal safe resection (ideally GTR) and CSI re-irradiation, with careful discussion of the potential side effects of these treatments.

**Key Points**

Re-irradiation is feasible and effective for locally recurrent pediatric ependymoma.
Repeat GTR is important to maximize PFS and OS for infratentorial recurrences.
3. CSI during RT2 may be associated with improved PFS.

**Importance of the Study**

This study describes significantly improved outcomes in progression-free survival (PFS) and overall survival (OS) when patients with locally recurrent ependymoma are treated with a gross total resection (GTR). We also describe a significant PFS benefit when surgery is followed by craniospinal irradiation (CSI), as compared to focal RT2. Few children developed treatment-related complications, suggesting that repeat radiation is a safe, feasible treatment option for recurrent disease, though longer-term follow-up is required. Our data will guide and inform clinical practice by supporting the importance of repeat maximal safe resection (ideally GTR) and re-irradiation (with CSI whenever possible) as treatment for recurrent pediatric ependymoma. Our large, multi-center dataset builds on previously reported single-institution series and suggests generalizability and safety of this treatment approach.

Ependymoma is the third most common intracranial tumor in children, comprising 6–10% of primary pediatric brain tumors.^1,2^ The upfront standard treatment for most patients with localized ependymoma is maximal safe surgical resection followed by focal radiation therapy (RT1; 54–59.4 Gy).^2^ Unfortunately, with the current standard of care, more than one-third of patients with ependymoma relapse,^3^ with one study demonstrating 5-year event-free survival of 68.5% despite upfront near or gross total resection with immediate post-operative conformal radiotherapy.^3^ Among all patients, it is estimated that 16% of children experience local failures while 11–12% experience distant failures.^4^ This number increases with further follow-up, with the propensity of ependymoma for late relapse being well documented.^5^ Studies suggest that survival for relapsed ependymoma with surgery alone is dismal,^6,7^ and chemotherapy unfortunately has not shown a strong benefit as salvage therapy.^8^

Retrospective studies strongly suggest that surgical resection followed by re-irradiation (RT2) confers a survival advantage for patients with recurrent ependymoma.^6,9–15^ However, these data are based on comparison to historical cohorts where the extent of surgical resection could have been less, compared to cohorts from more recent eras where patients received maximal safe resection followed by re-radiation. These studies also often vary in terms of number of patients, with heterogeneous treatments using different dose and fractionation regimens.^10,16–18^ The role of craniospinal irradiation (CSI) compared to focal reirradiation for local and distant recurrences also remains an area of ongoing controversy, especially as CSI can be associated with late side effects, such as neurocognitive impairment, hearing loss, endocrinopathy, and secondary malignancies. Furthermore, the efficacy of surgery and irradiation based on the timing of recurrence and reirradiation is an area that remains relatively unknown.

To better evaluate the role of RT2 in these patients, we completed a multi-institutional study of patients with locally recurrent ependymoma. This study aimed to evaluate clinicopathologic factors associated with overall (OS) and progression-free survival (PFS) in patients with locally recurrent pediatric ependymoma treated with a second course of radiation.

## Materials and Methods

This was a multi-institutional retrospective cohort study of patients with locally recurrent ependymoma, aged 21 years or younger (at the time of RT2) and treated with 2 courses of radiotherapy, between 1996 and 2018 at 6 North American cancer centers: Texas Children's Hospital (Houston, TX), MD Anderson Cancer Center (Houston, TX), University of Oklahoma Health Sciences Centre (Oklahoma City, OK), Arkansas Children's Hospital (Little Rock, AR), Cook Children's Hospital (Fort Worth, TX), and Princess Margaret Cancer Centre—University Health Network (Toronto, Canada). The Jimmy Everest Center for Cancer and Blood Diseases in Children (JEC) in Oklahoma (OK) and Princess Margaret Cancer Centre (PM) were coordinating centers, in collaboration with members of the Southern Pediatric Neuro-oncology Consortium (SOPNOC). In Toronto, children were jointly treated by the Hospital for Sick Children and PM. This multi-institutional study builds upon previous data reported by these 2 institutions and includes the patients previously reported.^19^ Locally recurrent ependymoma was defined as tumors that relapsed within the original tumor bed and within the initially irradiated field. Patients for whom the participating institution was not the primary oncology care provider, or those who received RT2 for neoplasms without evidence of ependymoma, were excluded. All patients with metastatic dissemination were also excluded. This study was approved by the respective institutional review boards at each participating institution.

Data were obtained from electronic medical records and clinical charts of patients corresponding to each participating institution. Histologic specimens from the United States, if available, were sent to a single core laboratory at the University of Oklahoma for H3K27me3 immunostaining to determine molecular subgroup.^20,21^ Molecular subgroup and copy number alterations for patients treated in Canada were determined locally using techniques previously described.^19,22^ Clinical data was collected using a uniform pre-determined data collection form with identifying information removed (Supplementary Figure 1). At first ependymoma recurrence, most patients underwent surgical tumor resection followed by RT2. The volume of RT2 was involved-field (focal) for a majority of patients, and CSI for some. In patients treated with stereotactic radiosurgery (SRS), doses were prescribed to the 50% isodose line. In patients treated with CSI as part of RT2 (all from PM), CSI was applied to the entire neuroaxis (without shielding applied to optic structures, brainstem, or cervical spinal cord) followed by a sequential focal boost to the site of locally recurrent tumor, as previously described.^19^ At PM, all focally recurrent patients with ependymoma were treated with CSI plus boost after 2012, following an institutional change of standard practice.^19^

Clinical factors and baseline characteristics were reported descriptively. Overall survival and PFS were measured from the first day of RT2; these outcomes were reported using the Kaplan–Meier method and compared using the log-rank test. Unless otherwise stated, PFS was defined as the time from RT2 to ependymoma recurrence. PFS1 was defined as time from RT1 to progression after RT1; PFS2 was defined as time from RT2 to progression after RT2. Univariate Cox regression was used to create models to evaluate factors associated with OS and PFS. Multivariable regression was not performed due to the small number of patients. The Wilcoxon rank-sum and signed-rank tests were used to compare continuous values for unpaired and paired data, respectively. Statistical analyses were completed using SAS 9.4 (SAS Institute, Cary, NC).

## Results

A total of 35 patients were eligible for inclusion. Baseline characteristics and radiation details are listed in Table 1. The median time from the first day of RT1 to initial local recurrence was 27.3 months (interquartile range [IQR] 16–43 months), from initial progression to RT2 was 1.1 months (IQR 1–2.6 months), and from the first day of RT1 to RT2 was 30 months (IQR 19–46 months, range 5.9–140.4 months). Twenty-two (63%) patients were treated at PM while the remainder (*n* = 13, 37%) were treated at other institutions in the United States. Nine patients (26%) received chemotherapy as part of their initial treatment.

**Table 1.** Baseline Characteristics for All Patients (*n* = 35)

Characteristic	Value
Age (years), median (range)	
Initial diagnosis	4.8 (0.8–16.3)
RT1 start	5.0 (1.2–16.4)
RT2 start	7.2 (2.4–22.3)
Female sex (%)	12 (34.3)
Initial site of ependymoma (%)	
Infratentorial	27 (77.1)
Supratentorial	8 (22.9)
Initial histologic grade (%)	
II	12 (34.3)
III	23 (65.7)
Molecular classification (%)	
PF-A	15 (42.9)
PF-B	1 (2.9)
RELA fused	3 (8.6)
Equivocal/unknown	16 (45.6)
Copy number alterations, infratentorial (%)	
1q intact	8 (100)
6q intact	8 (100)
Unknown	19
Pre-RT1 extent of resection (%)	
GTR	16 (45.7)
NTR	10 (28.57)
STR	9 (25.71)
RT1 dose (Gy), median (range)	55.8 (52.2-59.4)
RT1 modality (%)	
Photon—focal	31 (88.6)
Proton—focal	4 (11.4)
Pre-RT2 extent of resection (%)	
GTR	23 (65.7)
NTR	5 (14.3)
STR	5 (14.3)
No surgery	2 (5.7)
RT2 modality (%)	
Photon—focal	21 (60.0)
Photon—CSI + focal boost	7 (20.0)
Proton—focal	5 (14.3)
Photon—SRS	2 (5.7)
RT2 CSI dose (Gy), median (range)	23.4 (23.4-36.0)
RT2 dose (Gy), median (range)	54 (15-59.4)^a^

^a^Two patients received stereotactic radiosurgery of 15 Gy and 24 Gy.

All 35 patients received RT2 due to local in-field failure after RT1. No patient received concurrent chemotherapy with RT2. The most common RT1 doses were 54 Gy, in 16 patients, and 59.4 Gy, in 17 patients; the remaining 2 patients received 52.2 Gy and 55.8 Gy. The most common RT2 dose was 54 Gy, in 22 patients. Two patients treated with cobalt-60 stereotactic surgery (SRS) received 24 Gy and 15 Gy in a single fraction to local recurrences in the left vertex and 4th ventricle, respectively.

Median follow-up time from RT2 was 67 months (5.6 years), range 4–142 months. Median OS for all patients from RT2 was 65 months (95% confidence interval [CI] 34—not reached [NR]). Estimated OS after RT2 at 1-, 2-, and 5-years were 91.1% (95% CI 75.1–97.1), 81.2% (95% CI 62.6–91.1), and 52.5% (95% CI 31.9–69.5), respectively. Median PFS for all patients from RT2 was 33 months (95% CI 14–45.2 months). Estimated PFS after RT2 at 1-, 2-, and 5-years were 73.6% (95% CI 55.4–85.3), 55.8% (95% CI 37.7–70.5), and 29.7% (95% CI 14.6–46.6), respectively. Of the 21 patients who recurred after RT2, median time between RT2 to further recurrence was 14.0 months (IQR 10–26.7 months).

**Figure 1.**
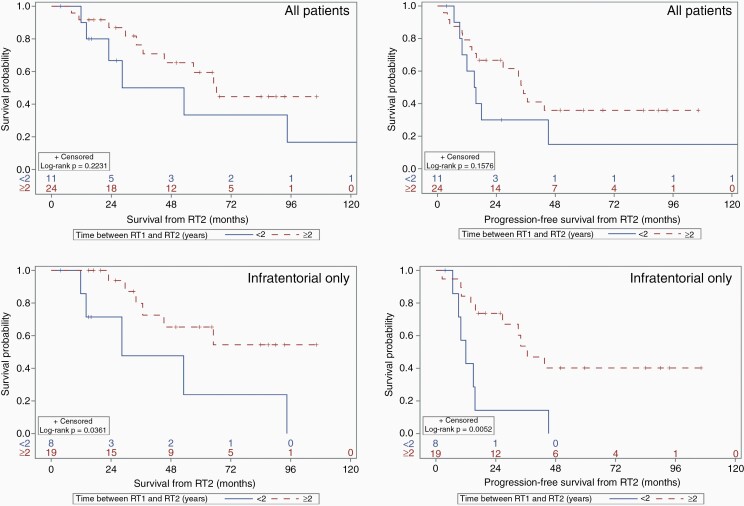
Overall (left) and progression-free survival (right) by elapsed time between initial radiation treatment and re-irradiation (in years) for all patients (top) and infratentorial tumors only (bottom). RT1 = initial course of fractionated radiation therapy. RT2 = second course of fractionated radiation therapy.

Figure 1 shows OS and PFS based on timing between RT1 and RT2, for all patients and for infratentorial tumors only. For all patients, median OS and PFS for those receiving re-irradiation within 2 years were 40.7 months (95% CI 11.8 months—NR) and 15.3 months (95% CI 6.7–45.2 months), respectively, while median OS and PFS for those who were re-irradiated after 2 years were 66.1 months (95% CI 36.7 months—NR) and 35 months (95% CI 14.0—NR), respectively; there was no statistically significant association. However, for infratentorial ependymomas, median OS and PFS for those requiring re-irradiation within 2 years were 28.4 months (95% CI 11.8–94.5 months) and 12 months (95% CI 6.7–15.7 months), respectively, while median OS and PFS for those who were re-irradiated after 2 years were not reached (95% CI 34 months—NR) and 36.6 months (95% CI 15.8 months—NR), respectively; these were statistically significant associations. For infratentorial tumors only, at least 2 years’ time interval between RT1 and RT2 was associated with a better OS (HR 0.29, 95% CI 0.09–0.98, *P* = 0.047) and PFS (HR 0.27, 95% CI 0.10–0.72, *P* = 0.009).

**Figure 2.**
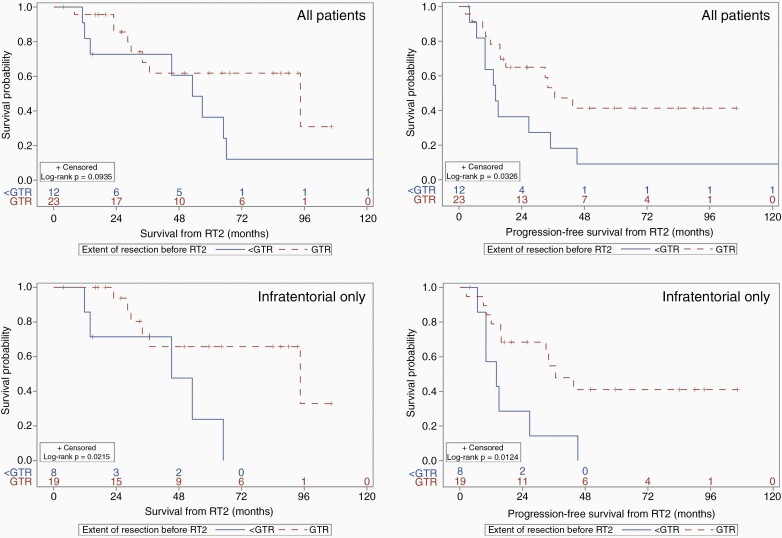
Overall (left) and progression-free survival (right) by extent of neurosurgical intervention at disease recurrence (pre-RT2) for all patients (top) and infratentorial tumors only (bottom). GTR = gross total resection. RT2 = second course of fractionated radiotherapy.

We subsequently explored the role of complete surgical resection at recurrence (Figure 2). For all patients receiving GTR at the time of recurrence (independent of the upfront extent of resection), median OS and PFS were 94.5 months (95% CI 29.6 months—NR) and 36.6 months (95% CI 15.8 months—NR), with a significant PFS benefit to GTR prior to re-irradiation (HR 0.41, *P* = 0.039). For infratentorial tumors, median OS and PFS for GTR prior to RT2 were 94.5 months (95% CI 34.0 months—NR) and 36.6 months (95% CI 15.7 months—NR), while that of non-GTR were 45.2 months (95% CI 11.8–65.0 months) and 14 months (95% CI 6.7–26.7 months), respectively. Gross total resection for recurrent infratentorial tumors was associated with a significant benefit for both OS (HR 3.9, 95% CI 1.12–13.6, *P* = 0.033) and PFS (HR 3.3, 95% CI 1.22–8.87, *P* = 0.018. Overall and progression-free survival, stratified by extent of initial resection prior to RT1, are reported in Supplementary Figure 2.

Patterns of failure and survival following focal re-irradiation vs. CSI as RT2 were also examined. Median follow-up of the CSI and focal RT2 cohorts were 59.5 months and 87.0 months, respectively. Median OS and PFS for those treated with focal re-irradiation were 56.9 months (95% CI 29.6–94.5 months) and 21.3 months (95% CI 12–36.6 months), while median OS and PFS for those treated with CSI were not reached. Furthermore, only one out of the 7 patients who received CSI-RT2 had disease recurrence (as a distant failure), compared to 20 out of 26 who recurred after receiving focal RT2 (Table 2). Local failures after RT2 were predominantly comprised of patients who had less than GTR prior to re-irradiation (6 patients with less than GTR, out of 8). Patients with WHO grade III histology (anaplastic) at diagnosis experienced 7 distant failures (out of 8), while only one patient with grade II histology experienced a distant failure. One out of the 2 patients who received SRS as RT2 experienced a local failure post-SRS.

**Table 2.** Post RT2 Patterns of Failure

RT2 Modality	Pre RT2 Extent of Resection			Post RT2 Pattern of Failure (*n*)	
	GTR	NTR	STR		
CSI (*n* = 7)	7			Distant	1
				**Total recurrences after CSI RT2**	**1**
SRS (*n* = 2)	No resection			Local	1
Focal (*n* = 26)	2	2	4	Local	8
	6	1		Distant	7
	1	2		Combined	3
	1			Other (lung mets)	1
				**Total recurrences after focal or SRS RT2**	**20**

Abbreviations: CSI, craniospinal irradiation; GTR, gross total resection; NTR, near total resection; STR, subtotal resection; SRS, stereotactic radiosurgery; RT2, second course of fractionated radiation therapy.

**Figure 3.**
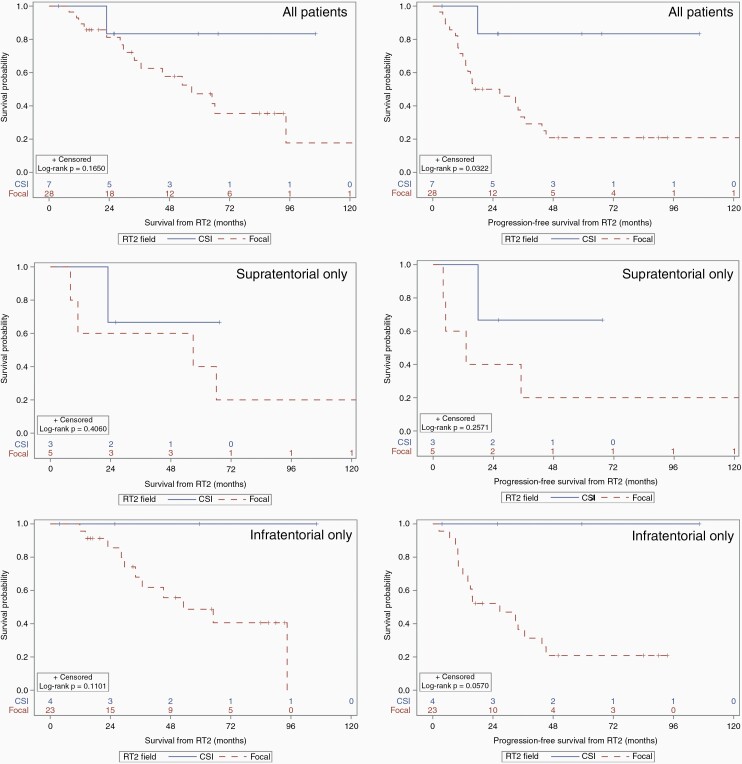
Overall (left) and progression-free survival (right) for patients by field of RT2 treatment for all patients (top), supratentorial tumors only (middle), and infratentorial tumors only (bottom). CSI = craniospinal irradiation. RT2 = second course of fractionated radiation therapy.

Furthermore, patients treated with CSI after recurrence experienced improved PFS (log-rank *P* = 0.032) but no statistically significant improvement in OS (*P* = 0.17), compared to those receiving focal re-irradiation (Figure 3). When considering infratentorial tumors only, there was no statistically significant OS or PFS benefit, though the latter did show a trend towards a benefit (log-rank *P* = 0.057; Figure 3). Similarly, among supratentorial tumors only, there was no detected benefit to CSI towards OS or PFS.

**Figure 4.**
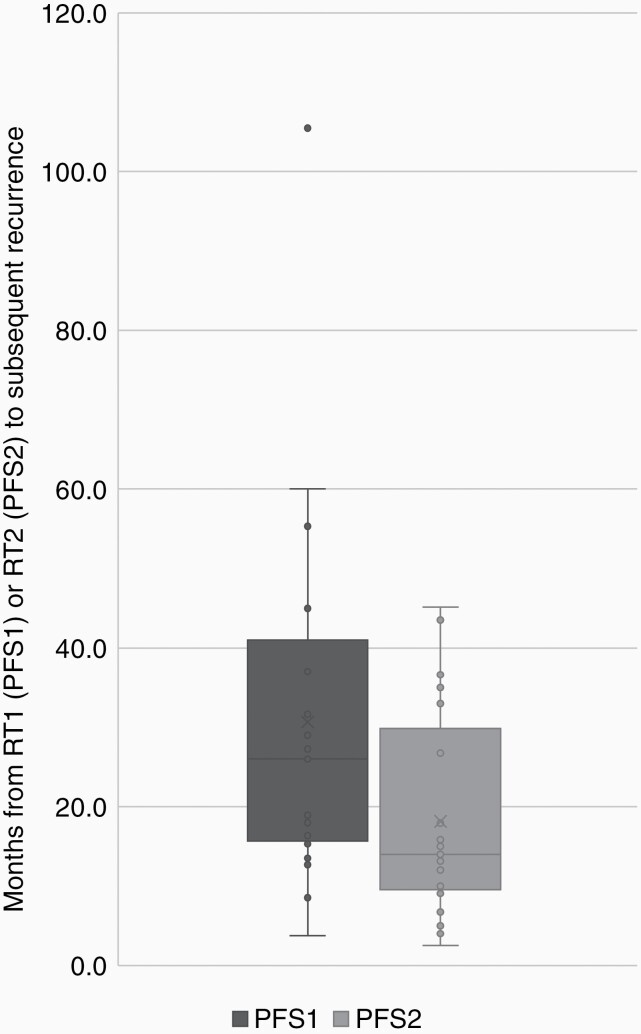
Progression free survival after RT1 and RT2 in patients with post-RT2 failure (*P* = 0.029). The boxes represent the first and third quartiles; the dark horizontal lines show medians, whiskers represent range (with outliers shown as points). PFS1 = PFS after first course of fractionated radiotherapy to recurrence after RT1; PFS2 = PFS after second course of fractionated radiotherapy to recurrence after RT2.

Within our cohort of patients, median PFS after RT1 (PFS1) and after RT2 (PFS2) were not statistically different: 27.3 months (95% CI 18.0–33.9 months) and 33 months (95% CI 14.0–45.2 months, *P* = 0.12), respectively (Supplementary Figure 3). When considering only the 21 patients who recurred after RT2, there was significantly shorter PFS2 compared to PFS1, with a median PFS2 and PFS1 of 14.0 and 26.0 months, respectively (*P* = 0.029, Figure 4). In comparison, PFS1 for patients who did *not* recur after RT2 was 31.7 months (vs. 26.0 months, *P* = 0.31).

Outcomes based on histological grade were also examined (Supplementary Figure 4), which although not statistically significant, suggest a trend to better PFS with CSI compared to focal radiation (*P* = 0.06), for both grade 2 and 3 ependymomas (Supplementary Table 1). Patterns of failure stratified by histology, RT2 field, and extent of pre-RT2 resection were also descriptively described (Supplementary Table 2) which demonstrate that few post-RT2 failures were observed after GTR of recurrent disease followed by CSI.

Factors associated with OS and PFS are listed in Supplementary Table 3. Sex, site of disease, and upfront chemotherapy were not significantly associated with improved OS and PFS, whereas GTR and grade were associated with PFS. Seven patients unfortunately recurred after RT2 and were treated with a third course of radiation, with 2 patients receiving a fourth course and 2 requiring a fifth course. The outcomes of 6 patients requiring *>*3 courses of radiation treated at PM have been described elsewhere.^19^ One patient, with 28.9 months between RT1 and RT2, developed grade 3 radiation necrosis after SRS-RT2, which resolved after 123 days with corticosteroids. An additional 3 patients developed radiation necrosis upon a third course of RT (RT3) or more, as determined by conventional MR imaging and symptoms (*n* = 2) or by MR spectroscopy (*n* = 2; Supplementary Table 4). All patients with necrosis eventually died of tumor recurrence (2 with persistent local failures, and 2 with distant failures). One additional patient developed an in-field secondary glioblastoma 18 months after focal RT2, and died 34 months after re-irradiation.

## Discussion

In this study, we present an analysis of 35 pediatric patients with locally recurrent ependymoma treated with 2 courses of radiotherapy across different North American pediatric cancer centers. We demonstrate that gross total resection at recurrence improves PFS, particularly for infratentorial primary tumors. Among recurrences of previously-irradiated infratentorial ependymomas, at least 2 years’ time interval between RT1 and RT2 was associated with better OS and PFS. Finally, re-irradiation with CSI improved PFS compared to focal re-treatment. Overall, this suggests that good surgical local control and re-irradiation with CSI are important components of salvage therapy.

Given the young age of many children with ependymoma and the potential long-term sequelae from CSI, if pursued, it is advisable to weigh the disease-control benefits and long-term sequelae of this treatment. This study was not able to evaluate the optimal dose of CSI, as both 23.4 Gy and 36 Gy were used. It may be prudent to reduce the total CSI dose delivered to recurrent disease in younger patients to minimize late toxicity, although further research in this area is required. Furthermore, in our cohort, the median follow-up time for those who received CSI RT2 was shorter than those who received focal RT2 (due to CSI re-irradiation only being introduced at PM starting in 2012). Therefore, longer follow-up for the CSI cohort is important to confirm the observed PFS difference over time.

### Surgical Resection

The improved prognosis associated with repeat surgery and re-irradiation for recurrent pediatric ependymoma are well described.^6,9–15^ While Eaton et al. have previously described improved OS with a maximal safe resection at recurrence,^23^ our data found that a GTR at the time of recurrence confers a PFS benefit only. Our dataset may be underpowered to evaluate OS, however. Interestingly, there has been mixed data published on this, with some studies describing a similar benefit on PFS,^12,13^ particularly in supratentorial tumors,^9,24^ while others have found no significant impact.^19^ Most recently, Adolph et al. described repeat GTR and NTR to be associated with a markedly improved 5-year survival, with an additional OS benefit of adjuvant re-irradiation only if a GTR or NTR was not achievable.^25^

In our subgroup analysis of infratentorial tumors, we found a significant benefit of repeat GTR on both PFS and OS. This is consistent with both the recurrent literature described above, as well as the upfront ependymoma literature; numerous studies have found that the extent of upfront surgical resection remains one of the most powerful prognostic factors for posterior fossa tumors.^3–5,26,27^ Therefore, it is appropriate to believe that a similar benefit may be applicable to repeat resection for infratentorial disease recurrence. Further studies to analyze infratentorial ependymomas are required and ongoing, given the documented differences in tumor biology compared to other molecular subtypes.^28,29^

Overall, it is clear that surgical resection remains critical in management of recurrent ependymoma. Our data demonstrate that attaining GTR at recurrence would allow the patient to remain progression-free for longer periods of time.

### Time to Radiation Re-treatment

Our analysis demonstrated that at least a 2-years' time interval between RT1 and RT2 was associated with improved PFS and OS among infratentorial tumors. This likely reflects more aggressive biology among tumors that recur quickly, thus needing shorter-interval re-treatment. However, data specific to interval RT1–RT2 time is limited, although it appears timing between radiation treatments is not necessarily associated with the development of radiation necrosis.^13,19^ Previous studies describing time between RT1 and local or distant recurrences (that were subsequently treated with RT2) have shown that a shorter interval between RT1 and recurrence was associated with reduced OS, and along with improved OS and PFS if the time to recurrence was greater than 4 years.^13^ Future, larger studies to investigate the timing of disease recurrence and re-treatment are likely to be beneficial, as one would hypothesize that delayed recurrence is likely associated with less aggressive disease and potentially longer duration of response to re-treatment.

### PFS1 vs. PFS2

In our analysis, PFS1 and PFS2 were not statistically different. This finding contrasts with previous studies of a similar sample size from Lobon et al. and Merchant et al., both of whom demonstrate prolonged PFS2 compared to PFS1.^12,15^ A larger study from Bouffet et al. also reveals conflicting data, as they described a significantly longer 3-year PFS2 (61%) compared to PFS1 (25%).^6^ These differences may be due to the large number of locally recurrent patients treated with focal RT2, particularly since we (and other studies) have described a PFS benefit of CSI, compared to focal re-treatment. Furthermore, 76% (*n* = 16) of recurrences had anaplastic histology, which is a known negative prognostic factor.^30^ These factors likely contributed to the shortened PFS2 observed in our cohort and highlight important prognostic histological and treatment factors.

### Comparison to the Literature

Our data are congruent with the previously described benefits of re-irradiation with CSI.^13,15^ Tsang et al. have previously described that CSI (vs. focal reirradiation) is safe and effective for local failures.^19^ With the addition of locally recurrent patients treated with focal RT2 from the other institutions from the United States, the PFS benefit of CSI was retained, which continues to suggest that the option of CSI at the time of recurrence should be strongly considered. Even with further stratification by histological grade, RT2 field, and extent of pre-RT2 resection, there were no failures post-RT2 with CSI in grade 2 ependymoma, and only 1 failure in grade 3 tumors treated with CSI. Although the role of histologic grade remains controversial in pediatric ependymoma,^31^ our data (though descriptive) do suggest that disease control is improved with GTR and CSI reirradiation.

Furthermore, apart from one case of secondary glioblastoma after RT2, any major radiation-related complications (Ie. Radiation Necrosis) in our cohort was only observed after SRS (which we no longer use for recurrent ependymoma) or 3 or more radiation treatments (described elsewhere^19^), suggesting that fractionated RT2 (preferably delivered as CSI) may be relatively safer than initially thought.

Previous studies have also shown male sex to be a negative prognostic factor,^4,13,23,32^ although the exact biological mechanisms behind this remain unknown. Interestingly, our data did not show this. This may suggest that additional sex-independent factors, such as tumor-specific mutations, play a role when prognosticating ependymoma survival. For example, the gain of chromosome 1q or 6q loss has been described as a negative prognostic factors^33^ and predictors for risk of relapse.^22^ Unfortunately, we were unable to obtain complete molecular work-up (including 1q gain or 6q loss) for all patients and were unable to further analyze the data based on these molecular characteristics.

Although our study utilizes multi-institutional data, it has some limitations. While the PFS benefit of CSI was retained, these cases were limited in number and only available from PM in Toronto because CSI was not used in the other institutions for locally recurrent ependymoma. While this may ensure consistency of decision-making and treatment parameters among patients who received CSI, it nonetheless does reflect a need to evaluate generalizability of study findings across different institutions in future research. Although some molecular data was gathered, it remained unknown for one-third of patients, precluding further analysis by ependymoma subtype. Infratentorial and supratentorial primaries are molecularly distinct,^29^ which was the rationale for performing subgroup analyses; however, there were insufficient supratentorial tumors (*n* = 8) to robustly analyze that subgroup. Statistical power to detect some associations with clinical factors was also likely limited due to the small sample size. There is also potential heterogeneity with regards to surgical, radiation, and imaging techniques experienced by these patients, given the long time-period from which data was gathered (1996–2018), which could limit applicability in the current era. Attempts at collecting neurocognitive function, educational outcomes, and incidence of endocrinopathy or vasculopathy following re-irradiation were also made, but not available due to the retrospective nature of this study across 6 institutions. Due to the time period of the study, we were unable to collect digital dosimetric data (such as doses to the brainstem, or how targets were delineated at each institution) from all institutions for further analysis, and therefore unable to evaluate optimal clinical target volume margins.

### Future Directions

Unfortunately, pediatric ependymoma recurrence is common, and long-term prognosis remains poor. Several studies have investigated the use of stereotactic radiosurgery with mixed results,^4,16–18^ and numerous clinical trials evaluating the use of immunotherapy (NCT02359565, NCT02774421) or HLA restricted peptides (NCT01795313) have failed to change management. Given the specific patient population in question, there are unfortunately very few prospective studies examining the role of surgery, radiation, or chemotherapy for recurrent ependymoma—unfortunately, a promising prospective study (NCT02125786) closed early. To our knowledge, the only active trial that remains is NCT03206021, which is a phase I study of systemic therapy only (5-azacitidine and carboplatin), and not RT2. Therefore, until other prospective data becomes available, retrospective data remains the best source from which to guide treatment. While this inherently comes with limitations in data collection and interpretation, it is nonetheless informative for the pediatric neuro-oncology community. At this time, it appears that re-resection and re-irradiation remain the best available therapies for disease recurrence.

## Conclusions

Repeat maximal safe resection and re-irradiation should be offered as treatment for recurrent pediatric ependymoma. Taken together with the described PFS benefit of repeat GTR and CSI for locally recurrent disease, we suggest that pediatric patients with recurrent ependymoma should be treated with a maximally safe GTR followed by CSI whenever possible. Future work into the neurocognitive sequelae of CSI re-irradiation with longitudinal follow-up of patients and their tumor control outcomes are needed to confirm the benefits of this approach.

## Supplementary Material

vdab158_suppl_Supplementary_Material
