# Supplementary material for: Reevaluating surgery and re-irradiation for locally recurrent pediatric ependymoma—a multi-institutional study
Source: Neurooncol Adv. 2021 Nov 8;3(1):vdab158. doi: 10.1093/noajnl/vdab158 (PMC8694210; doi:10.1093/noajnl/vdab158)
Supplement: vdab158_suppl_Supplementary_Material [file vdab158_suppl_supplementary_material.docx]

**Supplementary Material**


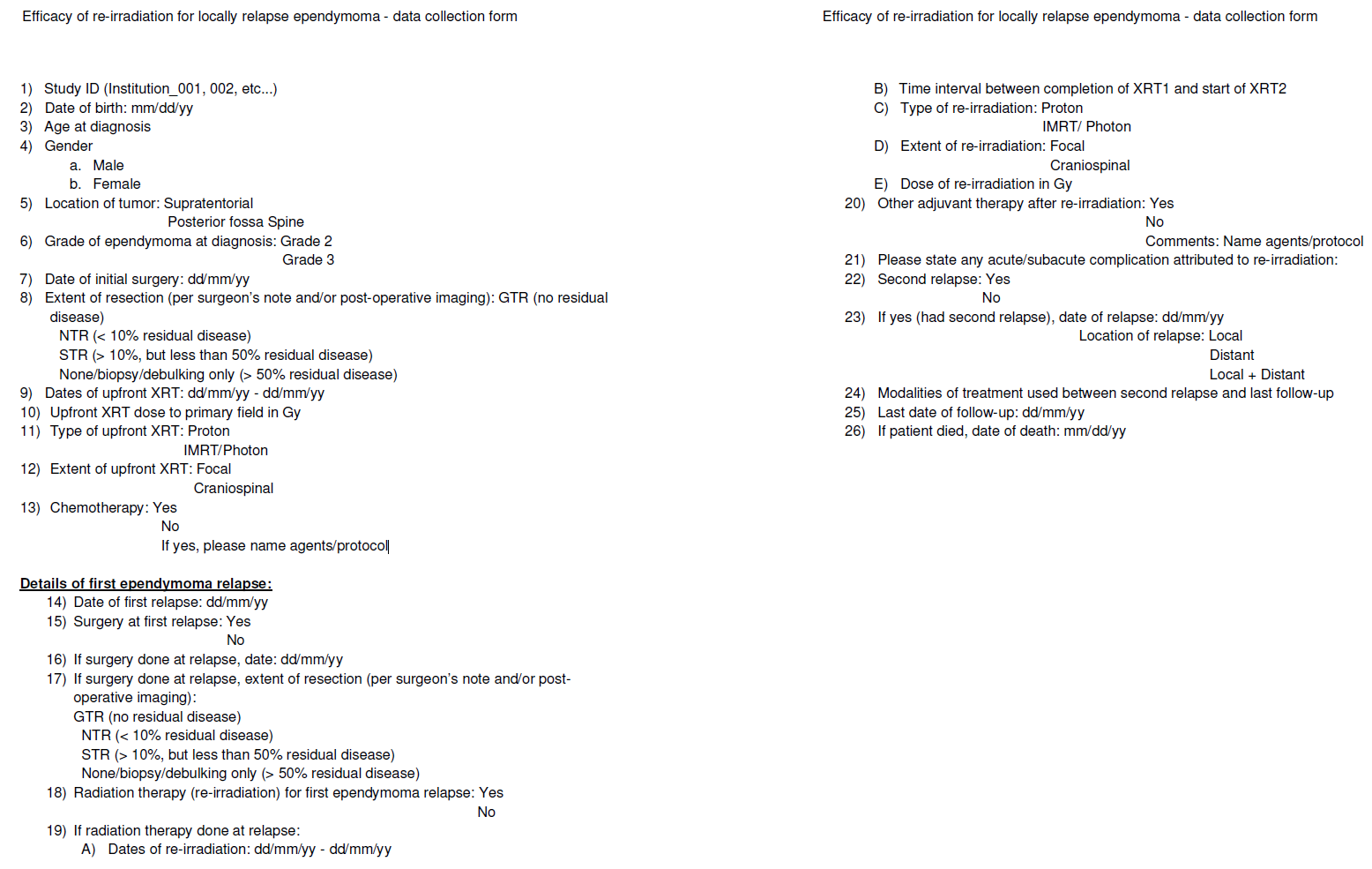


*Supplementary Figure 1. Case report form*


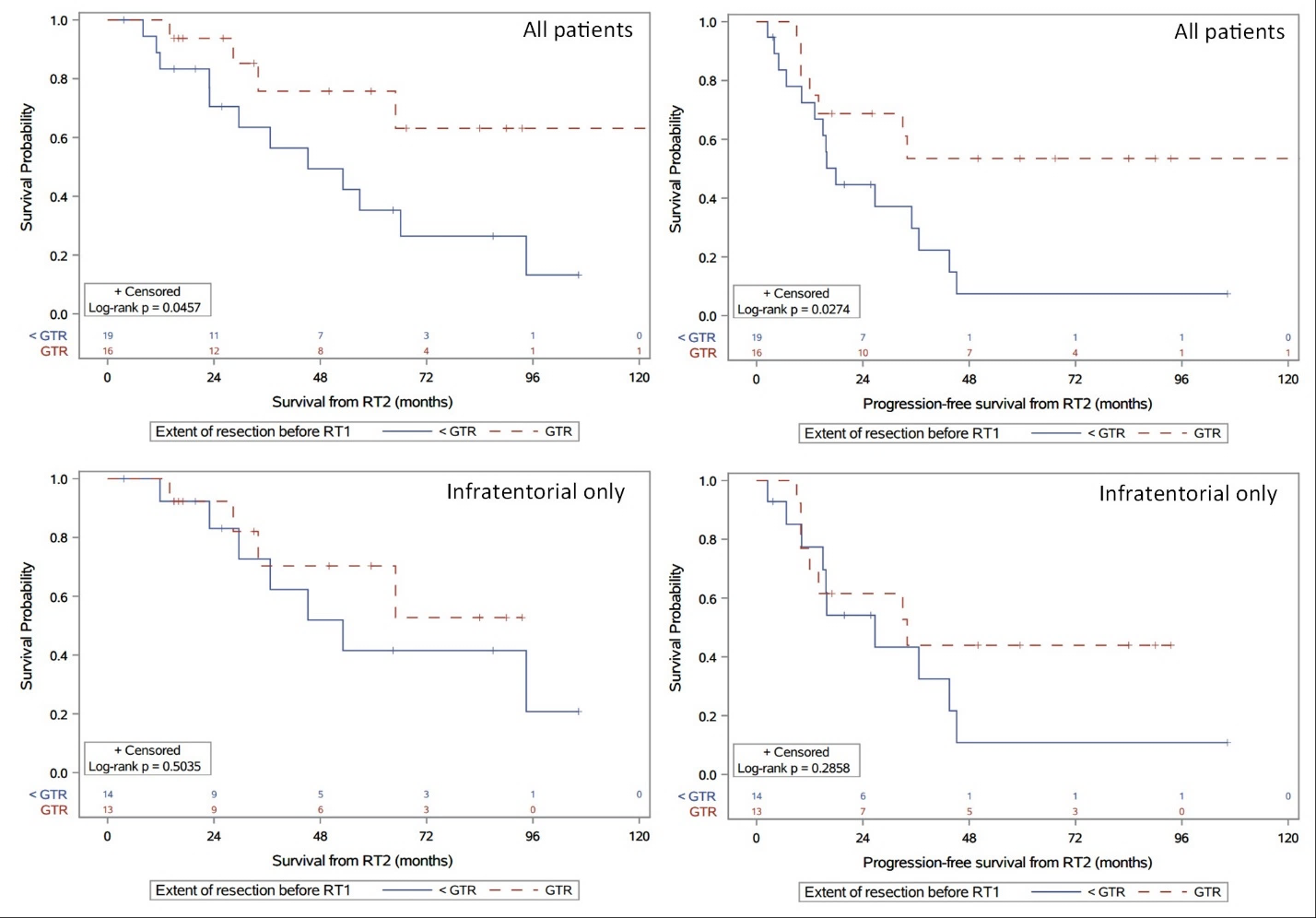


*Supplementary Figure 2.* *Overall (left) and progression-free survival (right) based on extent of neurosurgical intervention prior to radiotherapy at initial presentation (RT1) for all patients (top) and for infratentorial tumours only (bottom). GTR = gross total resection. RT1 = first course of fractionated radiotherapy.*

*
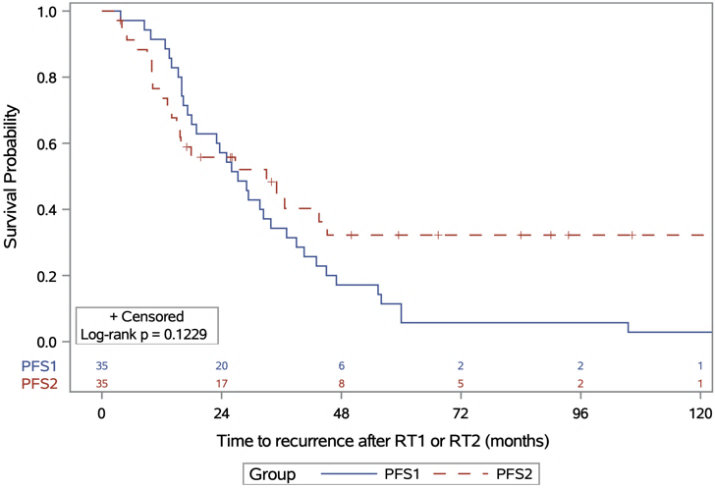
*

*Supplementary Figure 3. Progression free survival from RT1 to first recurrence (blue) and from RT2 to subsequent recurrence (red). RT1 = first course of fractionated radiation therapy; RT2 = second course of fractionated radiation therapy.*


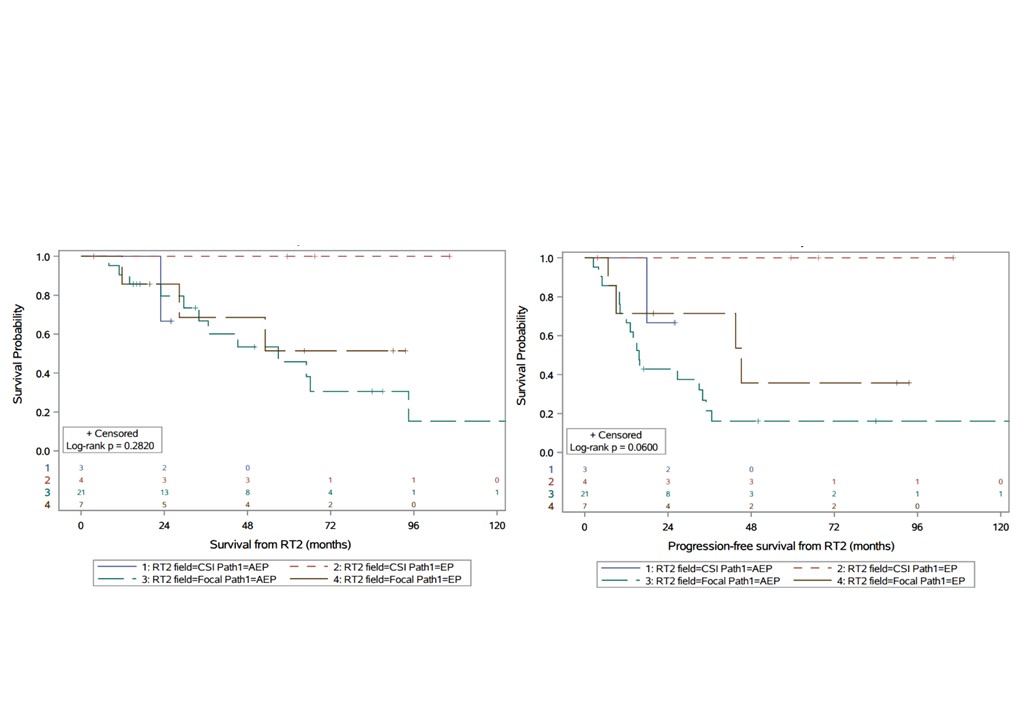


*Supplementary Figure 4. Overall and progression-free survival from RT2 stratified by RT2 field and histological grade. AEP = Anaplastic ependymoma (grade 3); CSI = craniospinal irradiation; EP – ependymoma (grade 2); Path1 = initial resection pathology; RT2 = second course of fractionated radiation therapy;*

| *Supplementary Table 1. Overall and progression free survival stratified by histological subtype and RT2 field.* | | | |
| --- | --- | --- | --- |
| **Histological grade** | **RT2 Field** | **Overall Survival (95% CI)** | **Progression Free Survival (95% CI)** |
| EP (Grade II) | Focal | Not reached | 45.2 months (6.7 – Not reached) |
|  | CSI | Not reached | Not reached |
| AEP (Grade III) | Focal | 56.9 months (29.6 – 94.4) | 15.7 months (19.2 – 34.0) |
|  | CSI | Not reached | Not reached |
| RT2 = second course of fractionated radiation therapy; CSI = craniospinal irradiation; AEP = anaplastic ependymoma (grade III); EP – Ependymoma (grade II) | | | |

| *Supplementary Table 2. Patterns of failure stratified by grade, RT2 field, and pre-RT2 resection.* | | | |
| --- | --- | --- | --- |
| **Histological grade** | **RT2 Field** | **Extent of pre-RT2 Resection** | **Post-RT2 Pattern of Failure** |
| EP - Grade II (n=12) | Focal (n=8) | GTR (n=5) | No local failures |
|  |  |  | Distant (n=2; 1 was lung mets) |
|  |  | <GTR (n=3) | Local (n=3) |
|  |  |  | No distant failures |
|  | CSI (n=4) | GTR (n=3) | No post-RT2 failures |
|  |  | <GTR (n=1) |  |
| AEP - Grade III (n=23) | Focal (n=18) | GTR (n=12) | Local (n=2) |
|  |  |  | Distant (n=5) |
|  |  |  | Combined local and distant (n=1) |
|  |  | <GTR (n=6) | Local (n=3) |
|  |  |  | Distant (n=1) |
|  |  |  | Combined local and distant (n=2) |
|  | CSI (n=3) | GTR (n=3) | No local failures |
|  |  |  | Distant (n=1) |
|  |  | <GTR (n=0) | N/A |
|  | SRS (n=2) | N/A | Local (n=1) |
|  |  |  | No distant failures |
| RT2 = second course of fractionated radiation therapy; CSI = craniospinal irradiation; AEP = anaplastic ependymoma (Grade III); EP – ependymoma (Grade II); GTR = gross total resection; SRS = stereotactic radiosurgery | | | |

| *Supplementary Table 3. Univariate Cox regression for factors associated with OS and PFS after RT2.* | | | | |
| --- | --- | --- | --- | --- |
| **Variable** | **Overall Survival** | | **Progression Free Survival** | |
|  | **HR (95% CI)** | **p-value** | **HR (95% CI)** | **p-value** |
| Female | 1.23 (0.44 – 3.4) | 0.69 | 1.46 (0.61 – 3.50) | 0.40 |
| Anaplastic pathology (Grade III) | 2.61 (0.74 – 9.23) | 0.14 | 3.36 (1.10 – 10.3) | 0.03 |
| Infratentorial tumour (vs. supratentorial) | 0.71 (0.25 – 2.07) | 0.54 | 0.96 (0.35 – 2.60) | 0.94 |
| Addition of chemotherapy to RT1 | 2.10 (0.58 – 7.58) | 0.26 | 2.20 (0.74 – 6.54) | 0.16 |
| GTR at initial diagnosis | 0.33 (0.11 – 1.03) | 0.057 | 0.37 (0.15 – 0.93) | 0.03 |
| GTR at initial recurrence | 0.44 (0.16 – 1.18) | 0.10 | 0.41 (0.18 – 0.96) | 0.04 |
| RT2 field (CSI vs. focal) | 0.26 (0.04 – 2.00) | 0.20 | 0.15 (0.02 – 1.12) | 0.064 |
| Treatment site (PM vs other) | 0.79 (0.34 – 2.95) | 0.67 | 0.71 (0.43 – 2.35) | 0.42 |
| HR = hazard ratio; NTR = near total resection; PM = Princess Margaret Cancer Centre; GTR = gross total resection; STR = subtotal resection; RT1 = first radiotherapy; RT2 = second course of fractionated radiation therapy | | | | |

| *Supplementary Table 4. Patients with radiation necrosis in recurrent ependymoma treated with re-irradiation. All patients died of subsequent tumour recurrence.* | | | | |  |
| --- | --- | --- | --- | --- | --- |
| **Patient** | **Necrosis Grade, Onset** | **Treatment** | **Necrosis resolution time** | **Survival after necrosis resolution** | **Pattern of failure after RT2** |
| 1 | Grade 1, Post SRS RT5 | Observation | 140 days | 3.8 years | Distant |
| 2 | Grade 2, Post SRS RT3 | Corticosteroids | 7 days | 1.1 years | Distant |
| 3 | Grade 3, Post SRS RT2 | Corticosteroids | 123 days | 3.3 years | Local |
| 4 | Grade 4, Post CSI RT3 | Bevacizumab | 72 days | 1.9 years | Local |
| CSI = craniospinal irradiation; RT*n* = *n*^th^ course of fractionated radiation therapy; SRS = stereotactic radiosurgery | | | | |  |
